# Prevalence and characterization of extended-spectrum beta-lactamases producing *Enterobacteriaceae* in healthy children and associated risk factors

**DOI:** 10.1186/s12941-016-0121-9

**Published:** 2016-01-29

**Authors:** S. M. Hijazi, M. A. Fawzi, F. M. Ali, K. H. Abd El Galil

**Affiliations:** Department of Pharmaceutical Sciences (Pharmaceutical Microbiology), Faculty of Pharmacy, Beirut Arab University, Beirut, Lebanon; Department of Pharmaceutical Microbiology, Faculty of Pharmacy, Alexandria University, Alexandria, Egypt; Department of Microbiology and Immunology, Infection Control, Faculty of Medicine, Ain Shams University, Cairo, Egypt; Department of Pharmaceutical Microbiology, Faculty of Pharmacy, Mansoura University, Mansoura, Egypt

**Keywords:** Extended-spectrum beta-lactamase, Children, TEM, SHV, CTX-M, CTX-9, Dairy products, Meat, Lebanon

## Abstract

**Background:**

Community acquired infections due to extended-spectrum beta-lactamase-producing *Enterobacteriaceae* (ESBL-PE) had been increased. The fecal flora of children in the community represents a huge potential reservoir for ESBLs which are located on highly transmissible plasmids. This study examined the prevalence of ESBL-PE fecal carriage, antimicrobial susceptibility pattern, possible risk factors, and characterized the genes encoding these ESBL enzymes in Lebanese children community.

**Methods:**

A total of 125 rectal swabs were taken from healthy children aged from 1 to 5 years. Detection of ESBLs was carried out using combination-disc method test and multiplex PCR. A questionnaire concerning child’s lifestyle and risk factors for ESBL carriage was illustrated.

**Results:**

Thirty-one of 125 participants (24.8 %) carried ESBL-PE. Regular consumption of meat, and chicken were significantly associated with high carriage rate of ESBL-PE, while dairy products (milk, yogurt, cheese) association was non-significant. Intimate hygiene habits were found also affecting the carriage rate. Multiple *bla* genes were the most common, 48.4 % (15/31) of ESBL-PE carried both *bla*_CTX-M_ and *bla*_TEM_, and 22.6 % (7/31) carried *bla*_CTX-M_, *bla*_SHV_, and *bla*_TEM_, 29 % (9) carried *bla*_CTX-M_ only. Concerning CTX-M-types, CTX-M-9 was the most predominant (24/31) and mostly in combination with CTX-M-15 type.

**Conclusion:**

High rate of colonization in healthy children with ESBL-PE was observed, regular consumption of dietary products from animal source (meat or chicken) were associated with this colonization in the community in non-hospitalized children. To our best knowledge it is the first study about regular consumption of dairy product as a risk factor for ESBL-PE community carriage, the first data about the carriage rate of ESBL-PE in community children in Lebanon and Middle East, and for the wide dissemination of CTX-M-9 type in this population.

## Background

*Enterobacteriaceae* carrying extended-spectrum β-lactamases (ESBLs) have emerged as significant pathogens. Such strains are resistant to multiple antimicrobial agents, and can be challenging to treat, as their therapeutic options are few [1]. Resistance to β-lactams in *Enterobacteriaceae* is primarily due to β-lactamases-mediated antibiotic hydrolysis; while an altered expression of efflux pumps and/or porins play only a minor role (2). Based on substrate specificities; the β-lactamases family is divided into four functional groups: penicillinases, ESBLs, carbapenemases, and AmpC-type cephalosporinases [2]. Of these, ESBLs, which can hydrolyze virtually all penicillins and cephalosporins, including the extended-spectrum cephalosporins, like cefotaxime or ceftazidime, comprise the largest and most prevalent group of enzymes [3]. Many ESBL producers are multi-resistant to non-β-lactam antibiotics, including fluoroquinolones and aminoglycosides [4], trimethoprim, tetracyclines, sulfonamides, and chloramphenicol as well as aminoglycosides, and this is often encoded by the same plasmids that determine the ESBL [5]. Consequently, effective antibiotic therapy for treating these infections is limited to a small number of drugs [6] such as carbapenems and thus increasing the chance of resistance to carbapenems among the *Enterobacteriaceae*. Of major concern is the coexistence of multiple ESBL, carbapenemase genes, and other antibiotic resistance determinants on mobile elements which may lead to the emergence of organisms with resistance to all antibiotic [2].

Rapid dissemination of *Escherichia coli* and *Klebsiella pneumoniae* isolates producing ESBLs widely in the community settings, resulted in both community-onset and hospital-associated infections on a global scale [7]. More than 200 types of ESBLs have been described in various species of the *Enterobacteriaceae* family and other non-enteric organisms, including *Pseudomonas aeruginosa* and *Acinetobacterspp.* These organisms produce variants of the TEM, SHV and CTX-M β-lactamases [8]. TEM- and SHV-type β-lactamases, mainly produced by *K. pneumoniae*, have spread throughout hospital settings, and CTX-M enzymes, mainly produced by *E. coli*, have become predominant in the community [9]. CTX-M enzymes were discovered in 1989 [10] but they did not become predominant over the other ESBL enzymes until the first decade of the twenty-first century during which an extraordinary spread of these enzymes was observed [11] in both hospital and community settings [12]. The *bla*_*CTX*-*M*-*15*_ gene are found mainly in *Enterobacteriaceae* and were recently named “plasmids of resistance responsible for outbreak” because of their capacity to acquire genes of resistance and to transfer among bacteria [13].

The community may thus represent a reservoir for ESBLs not detected in clinical isolates [14].

Most data on prevalence, risk factors and molecular characterization of ESBL -producing organisms are from studies in adult patients in both hospital and community settings, or from studies confined to hospitalized infants [15], only few data are available concerning the healthy pediatric populations. It is critical to better define the prevalence, risk factor, and molecular characterization of ESBLs-producing organism carried by children to adopt best-practice infection control measures and help in the appropriate choice of empirical antimicrobial coverage for infections in these populations.

The aim of this study is to investigate the prevalence and predisposing factors of intestinal carriage of ESBLs-producing *Enterobacteriaceae* (ESBL-PE) among Lebanese community children and to determine the molecular characterization of the resistance genes (*bla*_*TEM*_, *bla*_*CTX*_, *bla*_*SHV*_, *bla*_*CTX*-*M*-*2*_, *bla*_*CTX*-*M*-*9*_, and *bla*_*CTX*-*M*-*15*_).

## Methods

### Ethical clearance

The study protocols were approved by Institutional Review Board Committee of Beirut Arab University. Written informed consents were obtained from all patients parents (at least one parent of each child) before enrollment.

### Sample population and questionnaire

The study was performed between January 2013 and May 2013 in three different pediatric clinics in Lebanon. Only 125 healthy children population aged from 1 to 5 years old coming for vaccination or general checkup were chosen for this study. However, the children who were under antibiotic treatment within 5 days before the enrollment date were excluded from this study. A questionnaire was completed for each participant regarding name, age, gender, medical history (previous antibiotic or antacid intake, previous hospital admission), dietary habits (milk, yogurt, cheese, meat, or chicken consumption), and intimate hygiene habits.

### Bacterial isolation and ESBL detection and confirmation

Rectal swabs were taken by a sterile swabs moistened with sterile saline and were immediately plated onto MacConkey agar (Oxoid, UK) plates supplemented with 2 mg/l ceftazidime (used within 5 days of preparation), incubated at 37 °C for 24 h. When a sample showed positive growth on the selective medium, at least three colonies plus each distinct morphotype were selected for subsequent characterization. Bacterial identification was performed using Gram staining, biochemical testing (indole, methyl red, vogus-prauskaur, citrate, and urease), and API 20E system (bioMerieux,Marcy l’Etoile, France).

The isolates were first screened for ESBL production using ceftazidime, cefepime, cefotaxime, cefpodoxime, ceftriaxone, and aztreonam disks (Oxoid Ltd, Basingstone,UK) and then phenotypic confirmatory test was carried out by double disc synergy and the combination-disc method on Mueller–Hinton agar using ceftazidime, ceftazidime-clavulanic acid, cefotaxime, and cefotaxime-clavulanic acid. The organisms were considered to be ESBL producing when a ≥5-mm increase in a zone diameter for either antimicrobial agent tested in combination with clavulanic acid compared with the zone diameter of the agent when tested alone [16]. Then molecular analysis was done on all positive isolates screened.

### Antimicrobial susceptibility testing

The antimicrobial susceptibility testing of all ESBL-producers were examined by agar diffusion method according to the Clinical and Laboratory Standards Institute (CLSI) guidelines [16]. Antibiotic disc (Oxoid Ltd, Basingstone, UK): cefepime 30 µg, aztreonam 30 µg, imipenem 10 µg, meropenem 10 µg, gentamicin 10 µg, amikacin 30 µg, tetracycline 30 µg, ciprofloxacin 5 µg, levofloxacin 5 µg, norfloxacin 10 µg, nalidixic acid 30 µg, trimethoprim-sulfamethoxazole 12.5/23.75 µg, and ticarcillin 75 µg were used to determine resistance patterns of the collected isolates.

### Characterization of genes encoding ESBLs

Two conventional multiplex PCR was performed, the first one for the detection of TEM, SHV, CTX-M genes and the second for detection of CTX-M-2, CTX-M-9, CTX-M-15 encoding genes. Crude genomic DNA was extracted from the isolates by heat lysis. Briefly, one pure colony was suspended in 40 µl of sterile distilled water, and the cells were lysed by heating up at 95 °C for 5 min.

For the first PCR multiplex, suitable primers (Sigma-Aldrich) [17] each targeting selected region the *bla*_*TEM*_, *bla*_*SHV*_, and *bla*_*CTX*-*M*_ were used (Table 1).

**Table 1:** Primers for the *bla*_*TEM*_, *bla*_SHV_, and *bla*_CTX-M_ genes used in this study

Primers	Primer sequence 5′ to 3′	Size (bp)
TEM F TEM R	AGT GCT GCC ATA ACC ATG AGT G CTG ACT CCC CGT CGT GTA GAT A	431
SHV F SHV R	GAT GAA CGC TTT CCC ATG ATG CGC TGT TAT CGC TCA TGG TAA	214
CTX F CTX R	ATG TGC AGY ACC AGT AAR GT TGG GTR AAR TAR GTS ACC AGA	593

Amplification reactions were performed in a 25 μl volume in which 12.5 μl of PCR master mix 2× (Thermo scientific) reaction buffer containing 0.05 *Taq* DNA polymerase, 4 mM μ/μl MgCl2, 0.4 mM of each deoxynucleoside triphosphates mix dNTP (dATP, dCTP, dGTP, and dTTP), were mixed with 12.5 μl of DNA, primers, and H_2_O in the following manner; 0.5 μl TEM F, 0.5 μl TEM R, 1 μl of each remaining primers, (*SHV, CTX*-*M*) (10 μM/μl), 2.5 μl H_2_O, and 5 μl of the template DNA. Reactions were performed in a DNA thermal cycler (BIOER) under the following conditions: denaturation at 94 °C for 5 min followed by 30 cycles at 94 °C for 20 s, 61 °C for 30 s and 72 °C for 1 min with a final extension of 72 °C for 5 min [18].

For the second multiplex PCR, suitable primers each targeting selected region the *bla*_*CTX*-*M*-*2*_, *bla*_*CTX*-*M*-*9*_, and *bla*_*CTX*-*M*-*15*_ were used [17] (Table 2). Reactions were performed in a DNA thermal cycler under the following conditions: denaturation at 94 °C for 5 min followed by 30 cycles at 94 °C for 15 s, 56 °C for 15 s and 72 °C for 45 s with a final extension of 72 °C for 5 min.

**Table 2:** Primers for the *bla*_*CTX*-*M*-*2*_, *bla*_CTX-M-9_, and *bla*_CTX-M-15_ genes used in this study

Primers	Primer sequence 5′ to 3′	Size (bp)
CTX-M-2 F CTX-M-2 R	AAA CAG AGC GAG AGC GAT AAG GGG TAA AGT AGG TCA CCA GAA C	720
CTX-M-9 F CTX-M-9 R	GGA TTA ACC GTA TTG GGA GTT T GAT ACC GCA GAT AAT ACG CAG G	164
CTX-M-15 F CTX-M-15 R	CAC GTC AAT GGG ACG ATG T GAA AGG CAA TAC CAC CGG T	410

After PCR amplification, 2.5 μl of each reaction was separated by electrophoresis in 1.5 % agarose gel for 30 min at 100 V in 0.5× TBE buffer. DNA was stained with ethidium bromide (1 μg/ml) and the bands were detected using UV transilluminator (Cleaver Scientific Ltd).

### Statistical analysis

The data were analyzed using Yates corrected χ^2^ test. Chi square test was done to establish statistically difference in proportions for categorical data. Statistical significance was set as P values of <0.05 with confidence interval of 95 %. For multivariate logistic regression analysis, the two independent variables were dairy products and meat. For dairy products data were counted for consumer of any of the dairy products (milk, yogurt, or cheese). For meat variable data were counted for consumer for either animal meat or chicken collectively as meat. ESBL-PE carriage was the dependent variable. Statistical analysis was performed using Minitab program (Minitab 14 statistical software, PA, USA) and The Statistical Package for Social Sciences (SPSS, Version 20) program (IBM, Armonk, NY).

## Results

### Prevalence of ESBL-PE

One hundred twenty-five subjects participated in this study, 63 (50.4 %) were females and 62 (49.6 %) were males ranging in age from 1 to 5 years old. Of these participants, 31 (24.8 %) as shown in Table 3 were ESBL-PE carrier. Males had a higher colonization frequency (33.9 %) than did females (15.9 %) (P = 0.09).

**Table 3:** Factors associated with ESBL-PE fecal carriage in healthy children in Lebanese community

Characteristic	Total N (%)	ESBL+ N (%)	ESBL− N (%)	P value
	125 (100 %)	31 (24.8 %)	94 (75.2 %)	
Sex				0.009
Male	62 (49.6 %)	21 (67.7 %)	41 (43.6 %)	
Female	63 (50.4 %)	10 (32.3 %)	53 (56.4 %)	
Taking antibiotic during last 8 weeks				0.072
Yes	56 (44.8 %)	18 (58.1 %)	38 (40.41 %)	
No	69 (55.2 %)	13 (41.9 %)	56 (59.6 %)	
Hospital admission in past 12 months				0.019
Yes	23 (18.4 %)	10 (32.3 %)	13 (13.8 %)	
No	102 (81.6 %)	21(67.7 %)	81 (86.2 %)	
Taking antacid drug during last 8 weeks				0.072
Yes	11 (8.8 %)	5 (16.1 %)	6 (6.4 %)	
No	114 (91.2 %)	26 (83.9 %)	88 (93.6 %)	
Drinking milk				0.011
Regularly*	75 (60 %)	25 (80.6 %)	50 (53.2 %)	
Rare^^^ or never	50 (40 %)	6 (19.4 %)	44 (46.8 %)	
Drinking or eating yogurt				0.039
Regularly	77 (61.6 %)	23 (74.2 %)	54 (57.4 %)	
Rare or never	48 (38.4 %)	8 (25.8 %)	40 (42.6 %)	
Eating cheese				0.019
Regularly	74 (59.2 %)	23 (74.2 %)	51 (54.3 %)	
Rare or never	51 (40.8 %)	8 (25.8 %)	43 (45.7 %)	
Eating meat				0.02
Regularly	56 (44.8 %)	19 (61.3 %)	37 (39.4 %)	
Rare or never	69 (55.2 %)	12 (38.7 %)	57 (60.6 %)	
Eating chicken				0.03
Regularly	58 (46.6 %)	19 (61.3 %)	39 (41.5 %)	
Rare or never	67 (53.6 %)	12 (38.7 %)	55 (58.5 %)	
Intimate hygiene habit				0.07
Dry tissue or wipes	51 (40.8 %)	16 (51.6 %)	35 (37.2 %)	
Water + soap or water + tissue	74 (59.2 %)	15 (48.4 %)	59 (62.8 %)	
Sharing toilet				0.12
No	87 (69.6 %)	24 (77.4 %)	63(67 %)	
Yes	38 (30.4 %)	7 (22.6 %)	31(33 %)	

P value was calculated using Chi square test

* Regularly: was considered as 3 times or more per week

^^^Rare: was considered as 2 times or less per week

### Factors associated with ESBL-PE carriage

Subjects who used antibiotics or antacid in the last 8 weeks, had higher rate of ESBL-PE carriage than their counterparts (Table 3), though no significant differences between these variables, while subjects admitted to the hospital in the last 12 months, were significantly associated with high carriage rate 43.5 % (10/23) than their counterparts 20.6 % (21/102) with P = 0.019 (Table 3).

A univariate and multivariate logistic regression analysis were performed to test for regular consumption (3 times per week or more) of milk, yogurt, cheese, chicken, or meat and ESBL-PE carriage. As seen in Table 3 the univariate analysis for each predictor revealed significant impact (P < 0.05) for regular consumption of milk, yogurt, cheese, meat, or chicken. However the multivariate logistic regression analysis (Table 4) failed to reveal any of these dietary products consumed as a predictor of ESBL-PE carriage. To delineate the food type as dairy products or meat (meat or chicken) as a predictor, the analysis revealed the meat and chicken as significant (P < 0.02) predictor for ESBL-PE carriage while dairy products impact was non-significant (P > 0.2) (Table 5).

**Table 4:** Parameter estimates for multivariate logistic regression of different types of food as predictors for ESBL-PE carriage

Food type	P value	OR	95 % confidence interval for OR	
			Lower bound	Upper bound
Milk	0.130	3.276	0.705	15.215
Yogurt	0.994	0.995	0.243	4.078
Cheese	0.884	0.893	0.197	4.062
Meat	0.402	1.700	0.492	5.877
Chicken	0.905	1.080	0.307	3.792

OR, odds ratio of regular consumer of food versus rare or non-consumer for ESBL-PE positive compared to negative

**Table 5:** Parameter estimates for multivariate logistic regression of dairy products or meat and chicken as predictors for ESBL-PE carriage

	P value	OR	95 % CI for OR	
			Lower	Upper
Dairy	0.217	2.006	0.664	6.063
Meat/chicken	0.019	3.077	1.200	7.889

OR, odds ratio of regular consumer of food versus rare or non-consumer for ESBL-PE positive compared to negative

For intimate hygiene habits, participant who wash with water or with soap and water then dry with tissue have lower ESBL-PE carriage rate 20.3 % (15/74) compared to 31.4 % (16/51) for those using only dry tissue or wipes with P = 0.07, OR 1.8, 95 % CI for OR 0.94–3.42 (Table 3). Participant who used to share toilets (used public toilet) were found to have 18.4 % (7/38) carriage rate compared to 27.6 % (24/87) for those don’t share toilets and this was statistically insignificant.

### Antibiotic susceptibility data

**Fig. 1:**
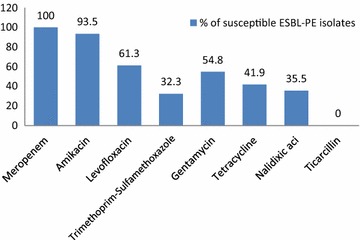
Antimicrobial susceptibility for 31 ESBL-PE carried by healthy Lebanese children

All isolates were resistant to azetreonam, cefepime, cefpodoxime, and ticarcillin. One hundred percent were susceptible to imipenem and meropenem, whereas 93.5 and 54.8 % were susceptible to amikacin and gentamicin respectively. Susceptibility to the quinolones tested (levofloxacin, ciprofloxacin) were 61.3 %. Furthermore, 41.9, 32.3, and 35.5 % of the isolates were susceptible to tetracycline, trimethoprim-sulfamethoxazole and nalidixic acid, respectively (Fig. 1).

### *bla gene* composition of ESBL-PE

**Fig. 2:**
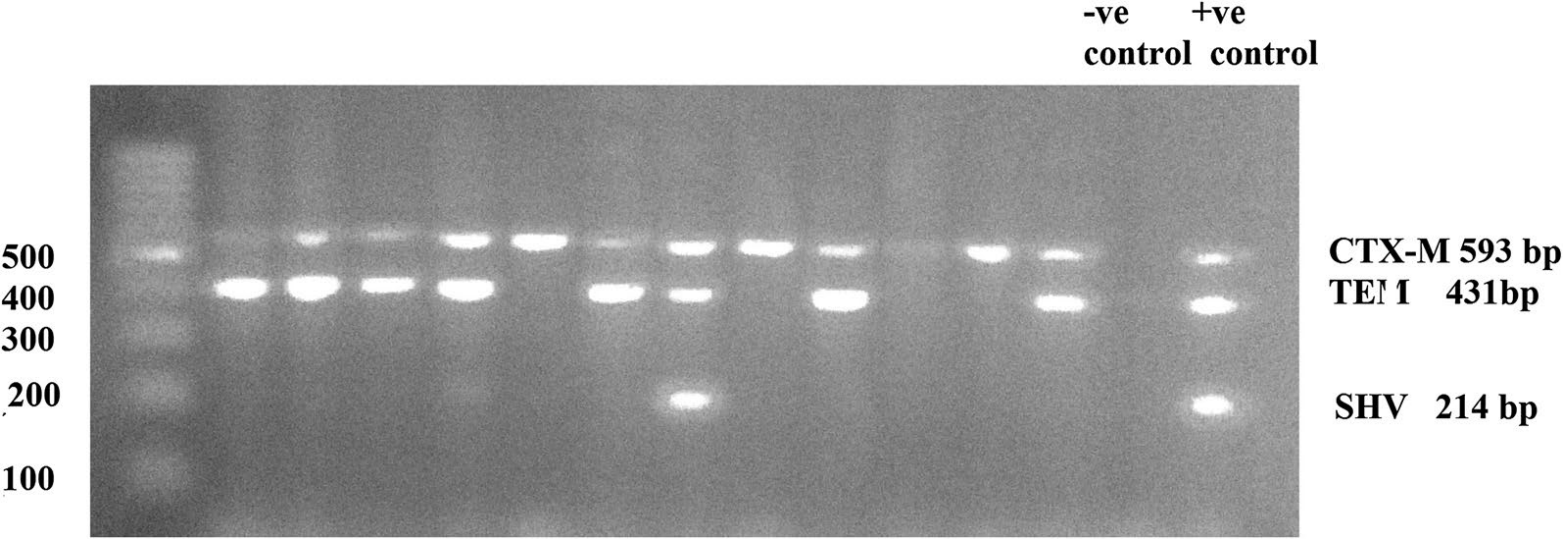
Agarose gel electrophoresis for PCR products obtained from ESBL-PE isolates with TEM, SHV, and CTX-M primers

Molecular characterization of 31 ESBL-PE among the pediatric isolates revealed that multiple gene producers were the most predominant 71 % (22/31), where CTX-M-type was produced by all isolates (31/31). Of the 31 CTX-M-producers isolates, 48.4 % (15/31) co-produced TEM, 22.6 % (7/31) co-produced SHV and TEM, and 29 % (9/31) produced CTX-M genes only (Fig. 2).

**Fig. 3:**
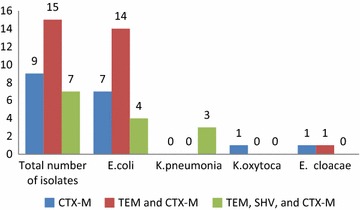
*bla* gene composition of the 31 ESBL-PE isolated from 125 rectal swabs obtained from healthy Lebanese children

The majority of ESBL-PE isolates recovered during the study were *E. coli* (n = 25), *K. pneumoniae* (n = 3), *Klebsiella* oxytoca (n = 1), and *Enterobacter cloacae* (n = 2) (Fig. 3). Fifty-six percent of isolated *E. coli* harbored *bla*_*CTX*-*M*_ and *bla*_*TEM*_ genes, 28 % harbored *bla*_*CTX*-*M*_ gene only, and 16 % harbored *bla*_*CTX*-*M*_, *bla*_*TEM*_, and *bla*_*SHV*_ genes. All *K. pneumonia* isolates harbored *bla*_*CTX*-*M*_, *bla*_*TEM*_, and *bla*_*SHV*_*genes*, where one isolated *E. cloacae* harbored *bla*_*CTX*-*M*_ gene and the other isolated one harbored *bla*_*CTX*-*M*_ and *bla*_*TEM*_ genes.

**Fig. 4:**
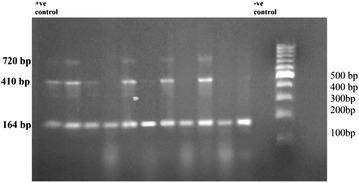
Agarose gel electrophoresis for PCR products obtained from ESBL-PE isolates with CTX-M-2, CTX-M-9, and CTX-M-15 primers

Molecular characterization of the 31ESBL-PE isolates carrying *bla*_*CTX*-*M*_ using specific primers for CTX-M-2, CTX-M-9, and CTX-M-15, revealed that CTX-M-9-type was the most common 77.4 % (24/31), and followed by CTX-M-15 [61.3 % (19/31)] and CTX-M-2 [29 % (9/31)] types (Fig. 4). Of these 24 CTX-M-9-producers, only 33.3 % (8/24) isolates produced CTX-M-9-type alone and the other isolates were co-producer of other types; 41.7 % (10/24) were CTX-M-15 co-producer, 25 % (6/24) were CTX-M-15 and CTX-M-2 co-producer. Two isolates produced CTX-M-15-type only, and one isolate produced both CTX-M-15-type and CTX-M-2-type (Table 6). The remaining four isolates (4/31) didn’t show any of these three CTX-M-types.

**Table 6:** Molecular characterization of 31 ESBL-PE isolates producing CTX-M-type obtained from rectal swabs of 125 healthy children in Lebanese community

CTX-M type	Total number of isolates N = 31	*E. coli* N = 25 (80.6 %)	*K. pneumonia* N = 3 (9.7 %)	*K. oxytoca* N = 1 (3.2 %)	*E. cloacae* N = 2 (6.5 %)
CTX-M-9	8 (25.8 %)	7 (28 %)	1 (33.3 %)	0 (0 %)	0 (0 %)
CTX-M-9 and CTX-M-15	10 (32.3 %)	9 (36 %)	0 (0 %)	1 (100 %)	0 (0 %)
CTX-M-9, CTX-M-15, and CTX-M-2	6 (19.3 %)	5 (20 %)	1 (33.3 %)	0 (0 %)	0 (0 %)
CTX-M-15	2 (6.5 %)	1 (4 %)	1 (33.3 %)	0 (0 %)	0 (0 %)
CTX-M-15 and CTX-M-2	1 (3.2 %)	1 (4 %)	0 (0 %)	0 (0 %)	0 (0 %)
CTX-M-2	0 (0 %)	0 (0 %)	0 (0 %)	0 (0 %)	0 (0 %)
CTX-M type other than 2, 9, 15	4 (12.9 %)	2 (8 %)	0 (0 %)	0 (0 %)	2 (100 %)

## Discussion

ESBL-producing organisms are increasingly common worldwide, they are recognized as important nosocomial pathogens in children, and are often associated with outbreaks [19]. Screening for intestinal carriage is crucial to predict the risk of ESBL infection, as the colon serves as a reservoir for extra-intestinal pathogenic *E. coli* [20].

This study was the first survey conducted in Lebanon on the intestinal carriage of ESBL-PE in the children community, and very rare studies were reported from Middle East. The prevalence was found to be 24.8 % which is high compared to 13.4, 2.7, and 2.9 % carriage rate in healthy pediatric in Libya, Portugal, and Sweden respectively [21–23]. Comparing this rate with fecal carriage in adult community in Saudi Arabia 12.7 % [24], in health care worker (HCW) in Egypt 21 % [25], and in adult community in 2005 in Lebanon 2.4 % [26], alarming high carriage rate was detected in this population that may serve as reservoir for dissemination of extra-intestinal ESBL-producing *E. coli* and *K. pneumoniae* and thus a source of infection in the community setting.

Males appear to have higher colonization rate than females. This observation is difficult to explain and needs to be further explored.

High carriage rate was in those individuals that used antibiotics or antacid in the last 8 weeks, or hospitalized in the last 12 months. Risk factors mentioned in the literature were prolonged hospital stays, living in nursing homes or long-term care facilities, underlying medical conditions, recent surgery, haemodialysis, and also prior use of antibiotics, particularly quinolones and third-generation cephalosporins, but also co-trimoxazole, aminoglycoside, and metronidazole [9].

Regarding antibiotic intake, although the selected children were healthy and not taking antibiotics (5 days before enrollment), it is noticed that 44.8 % of them have been taken antibiotics within last 8 weeks prior to being screened, this rate considered very high and reflects the high exposure of children to antibiotics and might explain the comparably high colonization rate. As known one of the main risk factors for the development of bacterial resistances is the increase of the consumption of several antibiotics [27]. In Lebanon there is no control measure and guidelines for antibiotic use in this age group. In addition, antibiotics are taken without medical prescription particularly in lower socioeconomic areas [28].

To the best of our knowledge, it is the first time to study regular consumption of dairy products (milk, yogurt, and cheese) and ESBL-PE carriage. In the present study, univariate analysis revealed significant association between regular consumption of dairy products, chicken, or meat with high ESBL-PE carriage rate. However analysis using multivariate logistic regression to test for the confounding factors, failed to show significant impact of any of these dietary products tested. This could be explained in part by the consumption of more than one type by most of the participants. Also the number of cases that consumed one type only is few to show the power of the test. This was obvious as gathering milk, yogurt and cheese as dairy products and meat and chicken together as meat showed significant association between meat and ESBL-PE colonization. A conclusion that meat is a predictor for ESBL-PE carriage compared to dairy products was also apparent by the insignificant impact of dairy products on ESBL-PE carriage, although both had higher incidence of ESBL positive isolates compared to control rare or never users. The odd ratio was 3 and 2 for meat and dairy products respectively. These dietary products are from animal source, and as known antibiotics are used for various purposes in agriculture and livestock production, for instance to boost growth or as therapeutic treatment and disease prophylaxis. *E. coli* and some other *Enterobacteriaceae* colonize the intestinal tract of both animals and humans, and recently the number of studies describing the prevalence of ESBL-PE in meat and raw milk has increased [29, 30]. Many studies had shown significant genetic similarities among ESBL-positive *E. coli* isolates from chicken meat and human according to mobile resistance elements, virulence genes and genomic back ground [31–33]. However other fewer contrasting observations showed that considerable differences in ESBL types between poultry and humans in Europe exist [34], and meat consumption is not related to ESBL transmission suggesting environmental sources such as water for drinking and food preparation [35]. There is no direct study between consuming dairy products and ESBL-PE carriage. A recent study in Tunisia isolated *E. coli* from cattle milk harbored the *bla*_*CTX*-*M*-*15*_ gene on an F2: A-:B-plasmid, a combination frequently found in humans [36]. Although the dissemination of resistance genes in *E. coli* may occur through multiple routes, our study show a potential threat posed by animal food products as sources for human ESBL-positive isolates [33]. Studies concerning ESBL-PE in dairy products and meat in the Lebanese market should be assessed to determine the impact of food products as reservoirs and disseminators of such strains through the food production chain to human.

Washing with water, or soap and water, the intimate area after urination, fecal evacuation, or diaper change might have tendency towards protective effect against ESBL-PE carriage compared to using only dry tissue or wipes, although P = 0.07, this might be further studied and explained.

Apart from a high rate of susceptibility to imipenem and amikacin, most isolates were resistant to other antimicrobials tested. ESBL-producing isolates were found highly resistant to nalidixic acid, tetracycline, gentamycin and to trimethoprim-sulfamethoxazole, and this result confirm carriage of multidrug-resistant ESBL-PE in this selected population of asymptomatic healthy children in Lebanon. This high carriage rate of ESBL-PE and associated resistance to aminoglycosides and trimethoprim-sulfamethoxazole, as well as high frequency of co-existance of fluoroquinolone resistance, increases the risk of infection with multi-resistant bacteria, and thus the need for usage of last resort antibiotics, such as carbapenems and colistin, in the treatment of common infections [6]. Majority of ESBL-PE carry multiple *bla* genes, where CTX-M-type was the most predominant. CTX-M ESBLs has been increased worldwide among *E. coli* and *Klebsiella spp*., this is the first study in the Lebanon that specifically determined the high prevalence of CTX-M-type ESBLs isolated from a healthy asymptomatic children, where CTX-M type beta-lactamases enzyme were produced by all isolates, and the majority of the isolates were found to be co-producers of the TEM gene, hence, TEM type was considered also a frequent disseminated beta-lactamases type. This was in contrast to a study in Egypt HCW where SHV was the predominant [25].This massive dissemination of CTX-M-type ESBLs in our result and its rapid and global spread almost reported everywhere could be referred as ‘CTX-M pandemic’ [37, 38], as the CTX-M-encoding plasmids are often transmissible by conjugation in vitro; this property explains the easy dissemination of *bla*_CTX-M_-harboring plasmids [39]. This dissemination of the CTX-M-type ESBLs is not restricted to the nosocomial setting but also involves the community. This phenomenon is acting to modify the epidemiology of ESBLs, whereas those enzymes were, previously, mostly restricted to the nosocomial setting [40], this may be correlated with food processing channel especially those from animal origin.

Molecular characterization of CTX-M-type ESBL revealed that the majority was CTX-M-9, followed by CTX-M-15 type. The greater number of isolates was co-producer of either 2 or 3 genes (*bla*_*CTX*-*M*-*9*_, *bla*_*CTX*-*M*-*15*_, *bla*_*CTX*-*M*-*2*_) together. CTX-M-9 was first reported from *E. coli* obtained from urine specimen from Spain and Brazil in1996 [41, 42], and recently one report from India [43]. Where CTX-M-type was predominant in *E. coli* obtained from Libyan children with diverse groups of CTX-M-1, CTX-M-15, and CTX-M-3 [21], major type was CTX-M-15 and no CTX-M-2 and CTX-M-9-types in *E. coli* isolate obtained from stool of Tehran children suffering from diarrhea [44], and CTX-M-1, CTX-M-15, and CTX-M-14 were predominant types of ESBL-PE obtained from French children rectal swabs [45].

This is the first study from Lebanon that demonstrates such a high prevalence of CTX-M-9 enzymes among *Enterobacteriaceae* strains isolated from community children. The *bla*_CTX-M-9_ was the predominant ESBL gene in this setting. Although CTX-M-15 are the most frequent CTX-M enzymes isolated in humans, animals, as well as in the environment, worldwide [46], and associated with the global CTX-M pandemic, but in our study CTX-M-9-type ESBL appears the most predominant. This is an indication that new mechanisms of resistance might be emerging in Lebanon.

## Conclusion

The carriage of ESBL-PE in young children in the Lebanese community is very high compared to the other countries. A significant correlation was found between this colonization rate and regular consumption of meat or chicken. Alarming multidrug resistance ESBL-PE is of great concern especially in this age group. Furthermore ESBL-PE multiple gene carriage of TEM, SHV, and CTX-M was the predominant, where CTX-M-9 and CTX-M-15 were the mostly spreader ESBL type.

## References

[1] Bradford PA (2001). Extended-spectrum beta-lactamases in the 21st century: characterization, epidemiology, and detection of this important resistance threat. Clin Microbiol Rev.

[2] Bush K (2010). Alarming beta-lactamase-mediated resistance in multidrug-resistant *Enterobacteriaceae*. Curr Opin Microbiol.

[3] Livermore DM (2012). Current epidemiology and growing resistance of Gram-negative pathogens. Korean J Intern Med.

[4] Livermore DM, Canton R, Gniadkowski M, Nordman P, Rossolini GM, Arlet G (2007). CTX-M: changing the face of ESBLs in Europe. J Antimicrob Chemother.

[5] Karisik E, Ellington MJ, Pike R, Warren RE, Livermore DM, Woodford N (2006). Molecular characterization of plasmids encoding CTX-M-15 beta-lactamases from *Escherichia coli* strains in the United Kingdom. J Antimicrob Chemother.

[6] Pitout JD (2010). Infections with extended-spectrum beta-lactamase producing *Enterobacteriaceae*: changing epidemiology and drug treatment choices. Drugs.

[7] Lewis JS, Herrera M, Wickes B, Patterson JE, Jorgensen JH (2007). First report of the emergence of CTX-M-type extended-spectrum beta-lactamases (ESBLs) as the predominant ESBL isolated in a US health care system. Antimicrob Agents Chemother.

[8] Paterson DL, Bonomo RA (2005). Extended-spectrum beta-lactamases: a clinical update. Clin Microbiol Rev.

[9] Pitout JDD, Laupland KB (2008). Extended-spectrum beta-lactamase-producing *Enterobacteriaceae*: an emerging public-health concern. Lancet Infect Dis.

[10] Bauernfeind A, Casellas JM, Goldberg M, Holley M, Jungwirth R, Mangold P (1992). A new plasmidic cefotaximase from patients infected with *Salmonella typhimurium*. Infection.

[11] Cantón R, Baquero F, Nombela C, Casslel GH, Gutierrez-Fuentes JA (2008). Epidemiology and evolution of β-lactamases. Evolutionary biology of bacterial and fungal pathogens.

[12] Cantón R, Coque TM (2006). The CTX-M β-lactamase pandemic. Curr Opin Microbiol.

[13] Coque TM, Novais A, Carattoli A, Poirel L, Pitout J, Peixe L (2008). Dissemination of clonally related *Escherichia coli* strains expressing extended-spectrum beta-lactamase CTX-M-15. Emerg Infect Dis.

[14] Valverde A, Coque TM, Sánchez-Moreno MP, Rollán A, Baquero F, Cantón R (2004). Dramatic increase in prevalence of fecal carriage of extended-spectrum beta-lactamase-producing *Enterobacteriaceae* during nonoutbreak situations in Spain. J Clin Microbiol.

[15] Chandramohan L, Revell PA (2012). Prevalence and molecular characterization of extended-spectrum β-lactamase producing Enterobacteriaceae in a pediatric patient population. Antimicrob Agents Chemother.

[16] Clinical and Laboratory Standards Institute. Performance standard for antimicrobial disk susceptibility testing: approved standards. 23rd ed. Wayne, PA: CLSI; 2013 (**document M100-S20**).

[17] Integrated DNA Technologies. http://eu.idtdna.com/site. Accessed 21 Sept 2013.

[18] Kim J, Jeon S, Lee B, Park M, Lee H, Lee J (2009). Rapid detection of extended spectrum β-lactamase (ESBL) for *Enterobacteriaceae* by use of a multiplex PCR-based method. Infect Chemother.

[19] Moissenet D, Salauze B, Clermont O, Bingen E, Arlet G, Denamur E (2010). Meningitis caused by *Escherichia coli* producing TEM-52 extended-spectrum beta-lactamase within an extensive outbreak in a neonatal ward: epidemiological investigation and characterization of the strain. J Clin Microbiol.

[20] Emori TG, Gaynes RP (1993). An overview of nosocomial infections, including the role of the microbiology laboratory. Clin Microbiol Rev.

[21] Ahmed SF, Ali MM, Mohamed ZK, Moussa TA, Klena JD (2014). Fecal carriage of extended-spectrum β-lactamases and AmpC-producing *Escherichia coli* in a Libyan community. Ann Clin Microbiol Antimicrob.

[22] Guimarães B, Barreto A, Radhouani H, Figueiredo N, Gaspar E, Rodrigues J (2009). Genetic detection of extended-spectrum beta-lactamasecontaining *Escherichia coli* isolates and vancomycin-resistant enterococci in fecal samples of healthy children. Microb Drug Resist.

[23] Kaarme J, Molin Y, Olsen B, Melhus A (2013). Prevalence of extended-spectrum beta-lactamase-producing *Enterobacteriaceae* in healthy Swedish preschool children. Acta Paediatr.

[24] Kader AA, Kamath KA (2009). Faecal carriage of extended-spectrum beta-lactamase-producing bacteria in the community. East Mediterr Health J.

[25] Bassyouni RH, Gaber SN, Wegdan AA (2015). Fecal carriage of extended-spectrum β-lactamase- and AmpC-producing *Escherichia coli* among healthcare workers. J Infect Dev Ctries.

[26] Moubareck C, Daoud Z, Hakime NI, Hamze M, Mangeney N, Matta H (2005). Countrywide spread of community- and hospital-acquired extended-spectrum betalactamase (CTX-M-15)-producing *Enterobacteriaceae* in Lebanon. J Clin Microbiol.

[27] Gómez J, García Vázquez E, Ruiz Gómez J (2008). Clinical relevance of bacterial resistance: a historical approach (1982–2007). Rev Esp Quimioter.

[28] Farah R, Lahoud N, Salameh P, Saleh N (2015). Antibiotic dispensation by Lebanese pharmacists: a comparison of higher and lower socio-economic levels. J Infect Public Health.

[29] Geser Nadine, Stephan Roger, Hächler Herbert (2012). Occurrence and characteristics of extended-spectrum β-lactamase (ESBL) producing *Enterobacteriaceae* in food producing animals, minced meat and raw milk. BMC Vet Res.

[30] Casella T, Rodríguez MM, Takahashi JT, Ghiglione B, Dropa M, Assunção E (2015). Detection of blaCTX-M-type genes in complex class 1 integrons carried by *Enterobacteriaceae* isolated from retail chicken meat in Brazil. Int J Food Microbiol.

[31] Overdevest I, Willemsen I, Rijnsburger M, Eustace A, Xu L, Hawkey P (2011). Extended-spectrum β-lactamase genes of *Escherichia coli* in chicken meat and humans, The Netherlands. Emerg Infect Dis.

[32] Kluytmans JA, Overdevest IT, Willemsen I, Kluytmans-van den Bergh MF, van der Zwaluw K, Heck M (2013). Extended-spectrum β-lactamase producing *Escherichia**coli* from retail chicken meat and humans: comparison of strains, plasmids, resistance genes, and virulence factors. Clin Infect Dis.

[33] Leverstein-van Hall MA, Dierikx CM, Cohen Stuart J, Voets GM, van den Munckhof MP, van Essen-Zandbergen A (2011). Dutch patients, retail chicken meat and poultry share the same ESBL genes, plasmids and strains. Clin Microbiol Infect.

[34] Wu G, Day MJ, Mafura MT, Nunez-Garcia J, Fenner JJ, Sharma M (2013). Comparative analysis of ESBL-positive *Escherichia**coli* isolates from animals and humans from the UK, The Netherlands and Germany. PLoS One.

[35] Königer D, Gastmeier P, Kola A, Schwab F, Meyer E (2014). Vegetarians are not less colonized with extended-spectrum-b-lactamase producing bacteria than meat eaters. J Antimicrob Chemother.

[36] Grami R, Dahmen S, Mansour W, Mehri W, Haenni M, Aouni M, Madec JY (2014). BlaCTX-M-15-carrying F2: A-: B-plasmid in *Escherichia coli* from cattle milk in Tunisia. Microb Drug Resist.

[37] Pournaras S, Ikonomidis A, Kristo I, Tsakris A, Maniatis AN (2004). CTX-M enzymes are the most common extended-spectrum β-lactamases among *Escherichia coli* in a tertiary Greek hospital. J Antimicrob Chemother.

[38] Yan JJ, Hsueh PR, Lu JJ, Chang FY, Shyr JM, Wan JH (2006). Extended-spectrum beta-lactamases and plasmid-mediated AmpC enzymes among clinical isolates of *Escherichia coli* and *Klebsiella pneumoniae* from seven medical centers in Taiwan. Antimicrob Agents Chemother.

[39] Baraniak A, Fiett J, Sulikowska A, Hryniewicz W, Gniadkowski M (2002). Countrywide spread of CTX-M-3 extended-spectrum beta-lactamase-producing microorganisms of the family *Enterobacteriaceae* in Poland. Antimicrob Agents Chemother.

[40] Rossolini GM, D’Andrea MM, Mugnaioli C (2008). The spread of CTX-M-type extended-spectrum β-lactamases. Clin Microbiol Infect Rev.

[41] Cantón R, Novais A, Valverde A, Machado E, Peixe L, Baquero F (2008). Prevalence and spread of extended-spectrum beta-lactamase-producing *Enterobacteriaceae* in Europe. Clin Microbiol Infect.

[42] Bonnet R (2004). Growing group of extended-spectrum beta-lactamases: the CTX-M enzymes. Antimicrob Agents Chemother.

[43] Roy S, Krishnan R, Mukherjee S, Schneiders T, Niyogi SK, Basu S (2013). Prevalence of ST131 virulence-associated strains among CTX-M-producing *Escherichia coli* in the gut of hospitalized neonates in India. Diagn Microbiol Infect Dis.

[44] Memariani M, Najar Peerayeh S, Zahraei Salehi T, Shokouhi Mostafavi SK (2015). Occurrence of SHV, TEM and CTX-M β-lactamase genes among enteropathogenic *Escherichia**coli* strains isolated from children with diarrhea. Jundishapur J Microbiol..

[45] Birgy André, Cohen Robert, Levy Corinne, Bidet Philippe, Courroux Céline, Benani Mohamed (2012). Community faecal carriage of extended-spectrum beta-lactamase-producing *Enterobacteriaceae* in French children. BMC Infect Dis.

[46] Lahlaoui H, Ben Haj Khalifa A, Ben Moussa M (2014). Epidemiology of *Enterobacteriaceae* producing CTX-M type extended spectrum β-lactamase (ESBL). Méd Mal Infect Rev.

